# Intravitreal dexamethasone implant (Ozurdex®) findings over time: ultrasound and ultra-widefield fundus photography

**DOI:** 10.1186/s40942-024-00625-6

**Published:** 2025-01-20

**Authors:** Gabriela Assumpção Brito Pereira Pellegrini, Arnaldo Furman Bordon, Norma Allemann

**Affiliations:** 1 Department of Retina-Vitreous and Ocular Ultrasound, Sorocaba Eye Hospital, Rua Nabeck Shiroma, 210, Jardim Emilia, Sorocaba, São Paulo/SP, 18031-060 Brazil; 2 Department of Ophthalmology and Visual Sciences, Federal University of São Paulo (UNIFESP), Rua Botucatu, 822, Vila Clementino, São Paulo/SP, 04023–062 Brazil

**Keywords:** Dexamethasone, Polymers, Intravitreal corticosteroid injection, Ocular ultrasound, Ultra-widefield

## Abstract

**Background:**

Ozurdex® (Allergan®, AbbVie Company, North Chicago, Illinois, EUA), is composed of 0.7 mg of dexamethasone, fused in a solid biodegradable PLGA polymer, whose degradation occurs naturally in the vitreous cavity, usually in six months after its application.

**Methods:**

In this study, we included patients aged ≥ 18 years with one or two eyes who had an indication for Ozurdex^®^ implants. Eyes submitted to Ozurdex^®^ application were evaluated in the first hour after the injection via transpalpebral contact B-scan ocular ultrasonography (Aviso® or Compact Touch^®^, Quantel^®^) and non-mydriatic ultra-widefield fundus photography (California^®^, Optos^®^) performed sequentially. The exams were executed using similar parameters and techniques, by the same ophthalmologist, after every 45 days, until the end of 180 days. The programed visits were the initial (tagged D0) and sequential (D45, D90, D135, and D180) visits, with a possible variance of seven days, before or after. The ultrasonographic Ozurdex^®^ findings evaluated were: non-quantitative: structure, height, reflectivity, artifact production, location, and movement; and quantitative: length and thickness. Ultra-widefield fundus photography parameters were: Ozurdex^®^ visualization, location, and structure.

**Results:**

The B-scan showed the implant initially, at the D0 visit, as a well-delimited and homogeneously highly reflective linear and continuous structure. On D45, Ozurdex^®^ implants presented with low internal reflectivity and irregularity in the limits. On D90, D135, and D180, reductions in the length and thickness progressively lessened, leading to the final appearance of a small highly reflective clust. Over time, all the implants presented reductions in length and thickness. The mean length at D0 was 7.42 ± 0.39 mm and at the final visit (D180) it was 1.50 ± 0.47 mm. The mean thickness at D0 was 0.77 ± 0.13 mm and at the final visit (D180) it was 0.44 ± 0.18 mm.

**Conclusions:**

Considering implant dimensions, the change in length over time was more evident than the change in thickness. In all the cases where visualization was possible, positive correlations with B-scan findings were found despite changes in patient position. These alterations evidenced in the Ozurdex® implant over time may be related to the degradation of the glucose polymer structure.

## Background

Intravitreal corticosteroid injections became the first-line treatment for many posterior segment diseases, offering the possibility to recover and preserve vision in challenging conditions. Ozurdex^®^ (Allergan^®^, AbbVie Company, North Chicago, Illinois, USA), a dexamethasone delivery system, is composed of 0.7 mg of dexamethasone, fused in a solid biodegradable poly lactic-co-glycolic acid polymer (Novadur^®^), whose degradation occurs naturally in the vitreous cavity, usually in a 6 month-period after application [1]. Hydrolysis and autocatalysis enable the slow release of dexamethasone, leading to the final products, glycolic and lactic acid [2]. It is known that the cylindric polymer’s structure initiates its decomposition internally, maintaining the external matrix wall with erosions, as demonstrated in vitro and in animal eyes [3]. Nevertheless, to the best of our knowledge, there is no study to evaluate Ozurdex^®^ polymer’s structure in the vitreous cavity of human eyes, sequentially. Our paper has examined eyes submitted to the Ozurdex^®^ implant, just after the application and in 45-day intervals, until a final period of 180 days, using multimodal evaluation with ocular ultrasonography and ultra-widefield fundus photography. The implant was detected to evaluate its localization, movement, and physical characteristics, such as the length, structure, thickness, and induction of artifacts. This is particularly important in patients whose implant visualization by fundoscopy is affected, such as those with small pupil sizes, opacities, and photophobia, and it enables the determination of the implant’s patterns during ocular movements in real time.

## Methods

Non-probability convenience sampling was used to select study participants. After obtaining informed consent from eligible participants, patients with one or two eyes who had an indication for Ozurdex^®^ implants were enrolled. Our inclusion criteria were as follows: eyes submitted to Ozurdex^®^ implant, clear media, age ≥ 18 years, and the exclusion criteria were as follows: complications following implantation, previous posterior pars plana vitrectomy, previous Ozurdex^®^ implant placement, and aphakia. Patients were not excluded for missing programmed visits as data collection continued in the next evaluation. Eyes submitted to Ozurdex^®^ application were evaluated in the first hour after the injection via transpalpebral contact B-scan ocular ultrasonography (Aviso^®^ or Compact Touch^®^, Quantel^®^) and non-mydriatic ultra-widefield fundus photography (California^®^, Optos^®^) performed sequentially. This visit was considered the initial one and tagged “D0”. The same exams were executed using similar parameters and techniques, by the same ophthalmologist, at intervals of 45 days, until the end of 180 days. The programmed visits were the initial (tagged D0) and sequential (D45, D90, D135, and D180) visits, with a possible variance of seven days, before or after. The ultrasonographic Ozurdex^®^ findings evaluated were: non-quantitative: structure, height, reflectivity, artifact production, location, and movement; and quantitative: length and thickness. Ultra-widefield fundus photography parameters were Ozurdex^®^ visualization, location, and structure.

### Data analysis

Quantitative data were presented as mean values with standard deviations for normally distributed data or median values with interquartile ranges for skewed data. Categorical data were presented as frequencies and percentages. The normality of data distribution was tested using the Shapiro–Wilk test. Differences between mean values were verified using the paired and unpaired Student’s t-test. The two-way ANOVA test was used to test differences between variance assumptions, followed by the Bonferroni post hoc test for statistically significant results. Differences with p ≤ 0.05 were considered statistically significant. For all data analyses, Microsoft Excel® V.2010 and SPSS® 26.0 software were used.

## Results

Twenty-five eyes of 24 patients were included in the study, and 16% of them were evaluated in all five visits proposed (D0, D45, D90, D135, and D180). All of the eyes completed the initial evaluation (D0) and 56% of them were evaluated in the last visit (D180). Twenty percent of them were present in four visits, 32% in three visits, and 12% in two visits, with none of them being necessarily consecutive.

Twenty percent of them came just for one visit, the first (D0), which was mandatory. Note that this study was conducted during the COVID-19 pandemic.

*Demographics* The mean age of our participants was 62.0 ± 12.3 years. The majority of patients were men (67%), and white (70.8%), and the most common indication for Ozurdex® treatment was diabetic macular edema (83.3%). Most of them were phakic (78.3%), and 21.7% were pseudophakic. None of the demographics or ocular characteristics cited had statistical significance in the analysis of the quantitative variants (length and thickness), as presented in Table 1.

**Table 1:** P value variation across visits in length and thickness, compared by demographics and ocular characteristics (age, gender, ethnicity and lens status)

	Length					Thickness				
	D0	D45	D90	D135	D180	D0	D45	D90	D135	D180
Age	0.29	0.10	0.30	0.30	0.24	0.76	0.10	0.09	0.06	0.41
Gender	0.06	0.40	0.31	0.55	0.68	0.96	0.39	0.92	0.35	0.87
Ethnicity	0.74	0.90	0.41	0.44	0.51	0.68	0.63	0.77	0.65	0.64
Lens status	0.20	0.15	0.32	0.36	0.53	0.06	0.66	0.27	0.42	0.28

*B-scan Qualitative Findings* All Ozurdex^®^ implants remained detectable in the vitreous cavity when the exam was performed, despite the possibility of early degradation. None of them was fragmented.

**Fig. 1:**
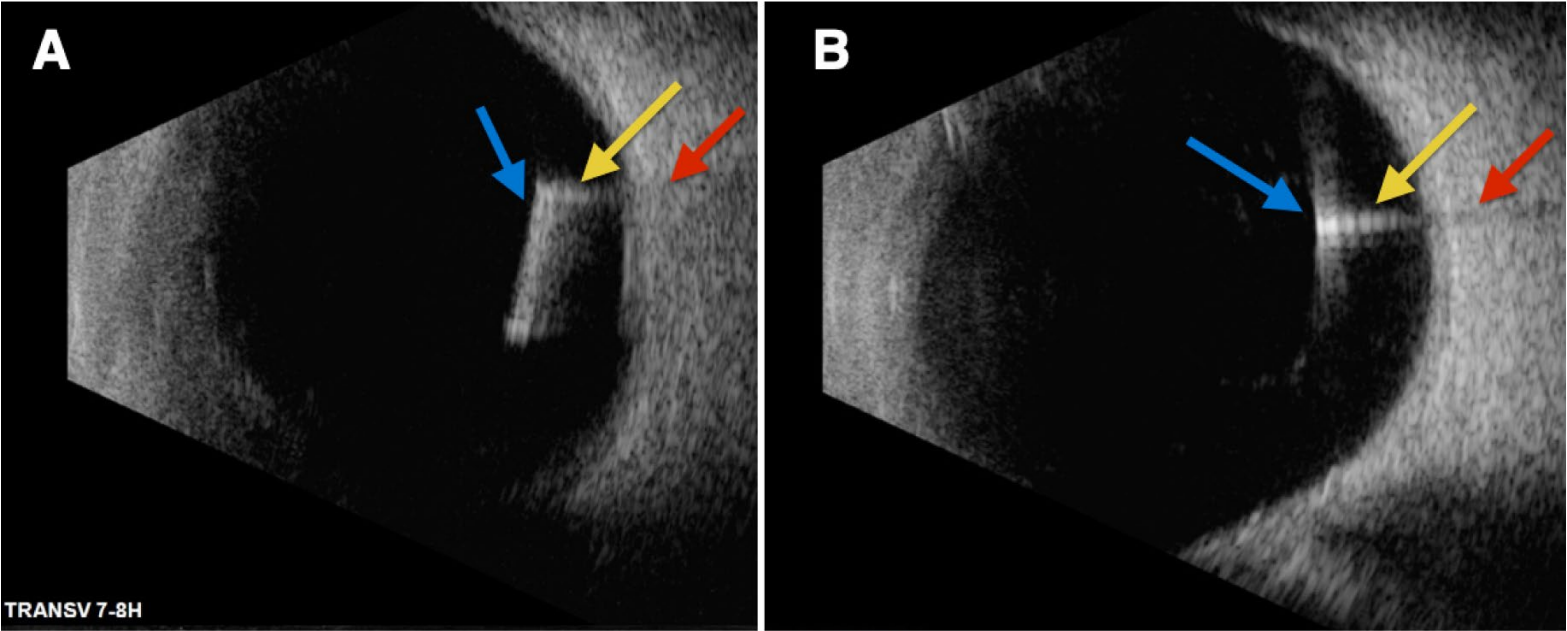
Ultrasound B-scan images of the same Ozurdex^®^ implant (blue arrows) in D0 visit, on total extension (**A**) and segmented (**B**). It is possible to evidence artifacts of reverberation in the vitreous cavity (yellow arrows) and shadowing of the eye wall (red arrows)

The B-scan showed the implant initially, at the D0 visit, as a well-delimited and homogeneously highly reflective linear and continuous structure, promoting artifacts of reverberation in the vitreous cavity and shadowing of the eye wall (Fig. 1A and B).

**Fig. 2:**
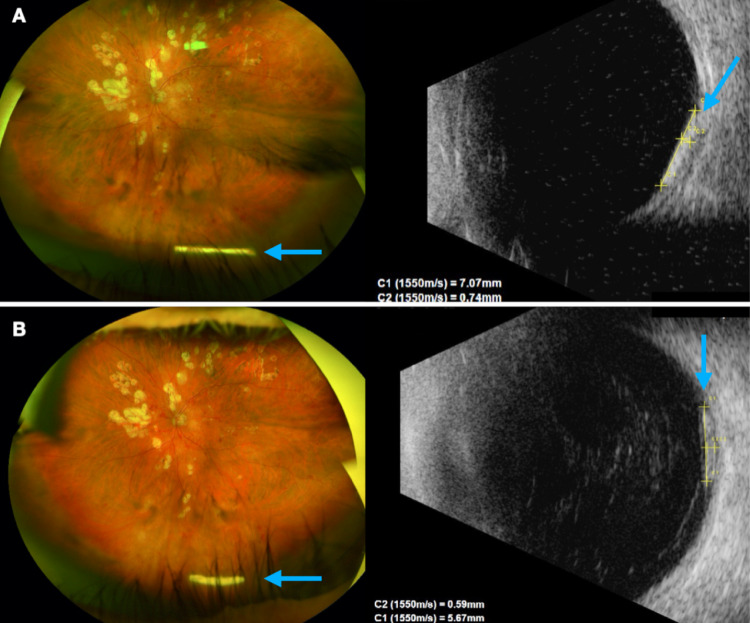
Ultra-widefield fundus photography (left) and B-Scan (right) showing the same Ozurdex^®^ implant (blue arrows) located directly on the retinal surface on D0 (**A**) and D45 (**B**) visits. It is evident the reduction of implant’s length (7.07 mm to 5.67 mm) and thickness (0.74 mm to 0.59 mm)

Ninety-two percent of them were located in the vitreous cavity, following its movement, and just two implants were resting directly on the retinal surface in all the visits (Fig. 2A and B).

**Fig. 3:**
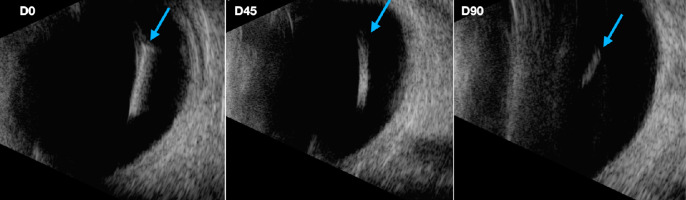
B-Scan of the same Ozurdex^®^ implant (blue arrows) on serial evaluation. Images showing reduction in length and thickness, lowering of internal reflectivity and the implant’s borders

On D45, Ozurdex^®^ implants presented with internal low reflectivity and irregularity in the limits (Fig. 3).

**Fig. 4:**
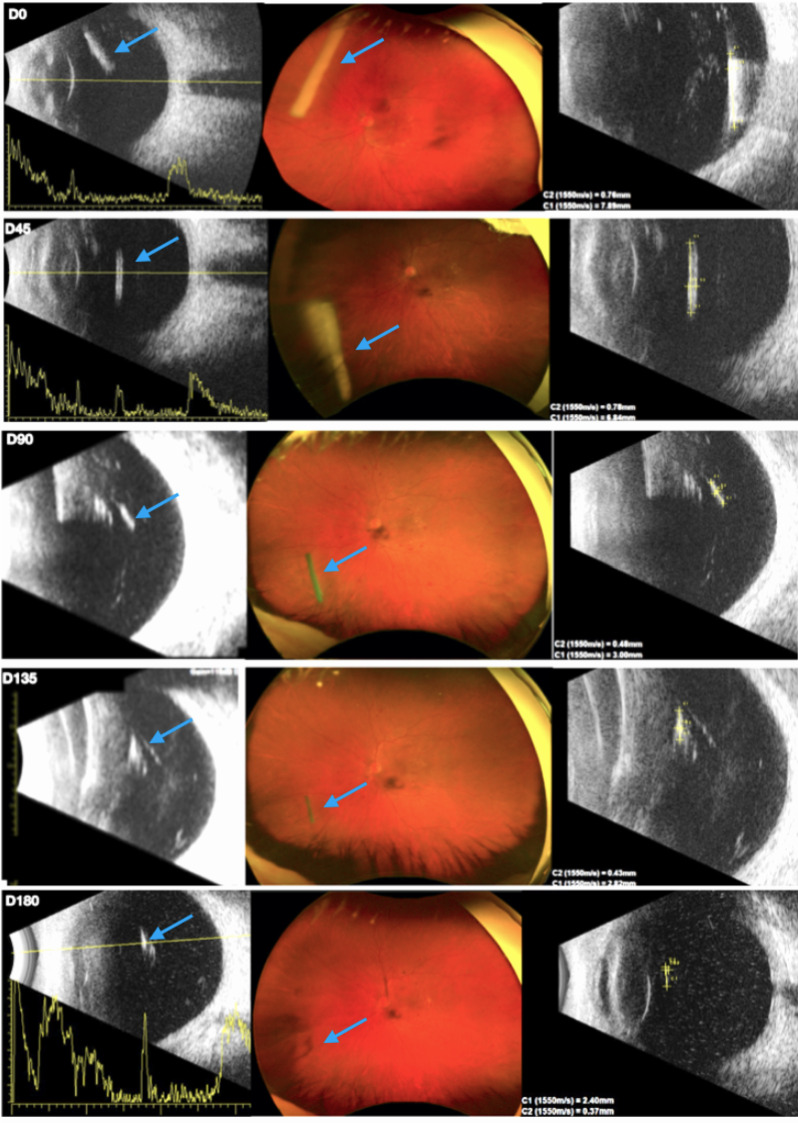
B-Scan (left and right) and Ultra-wiidefield fundus photography (middle) showing Ozurdex^®^ implant (blue arrows) characteristics and metrics over time, in the same eye (D0, D45, D90, D135 and D180)

On D90, D135, and D180, reductions in the length and thickness progressively lessened, leading to the final appearance of a small highly reflective clust (Fig. 4).

**Fig. 5:**
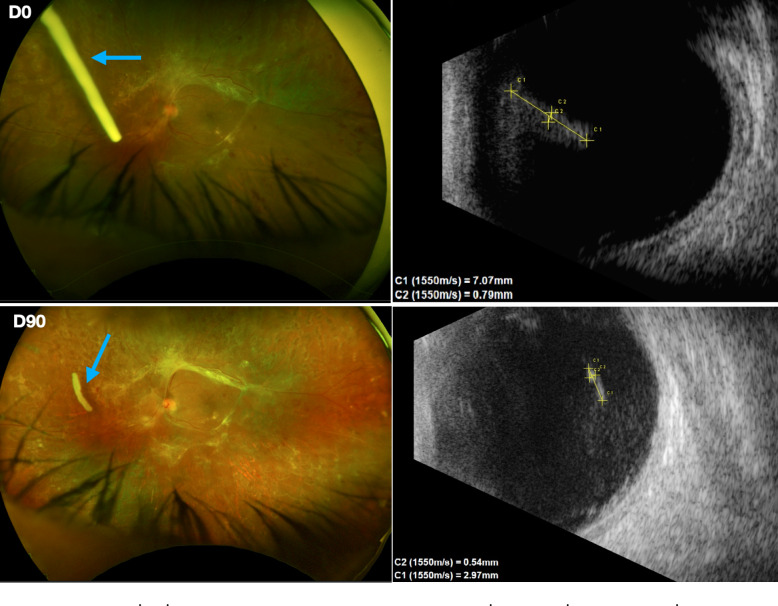
Ultra-widefield fundus photography (left) and B-Scan (right) showing Ozurdex^®^ implant (blue arrows) with curvilinear appearance on D90 visit

Twenty-eight percent of the implants became curvilinear (Fig. 5), leading to a false smaller final length since the measure was taken considering the implant’s limits.

*B-scan Quantitative Findings* B-scan gain, probe direction, and magnification were dynamically adjusted to scan the implant in its total extension for measures of thickness and length. Over time, all the implants presented reductions in these parameters, particularly in length. Ozurdex^®^ B-scan measurements varied over time. The mean length values were: D0 was 7.42 ± 0.39 mm, D45 and at the final visit (D180) it was 1.50 ± 0.47 mm. The mean thickness at D0 was 0.77 ± 0.13 mm and at the final visit (D180) it was 0.44 ± 0.18 mm. Considering implant dimensions, the change in length over time was more evident than the change in thickness, with the most noticeable changes occurring at the first evaluation (P = 0.001), as illustrated in Table 2. It is important to note that the pre-use dimensions of the Ozurdex^®^ implant are approximately 6.00 mm in length and 0.46 mm in thickness.

**Table 2:** Ozurdex® mean value, standard deviation and P value analysis in length and thickness in serial evaluation

	Length			Thickness		
	Mean value (mm)	Standard deviation (mm)	P value	Mean Value (mm)	Standard Deviation (mm)	P value
D0	7.42	± 0.39	0.001	0.77	± 0.39	0.001
D45	6.20	± 0.47	0.317	0.66	± 0.47	0.035
D90	3.12	± 0.73	> 0.999	0.53	± 0.73	0.008
D135	2.54	± 0.66	> 0.999	0.52	± 0.66	> 0.999
D180	1.50	± 0.47	> 0.999	0.44	± 0.47	> 0.999
mm (millimeters)						

**Fig. 6:**
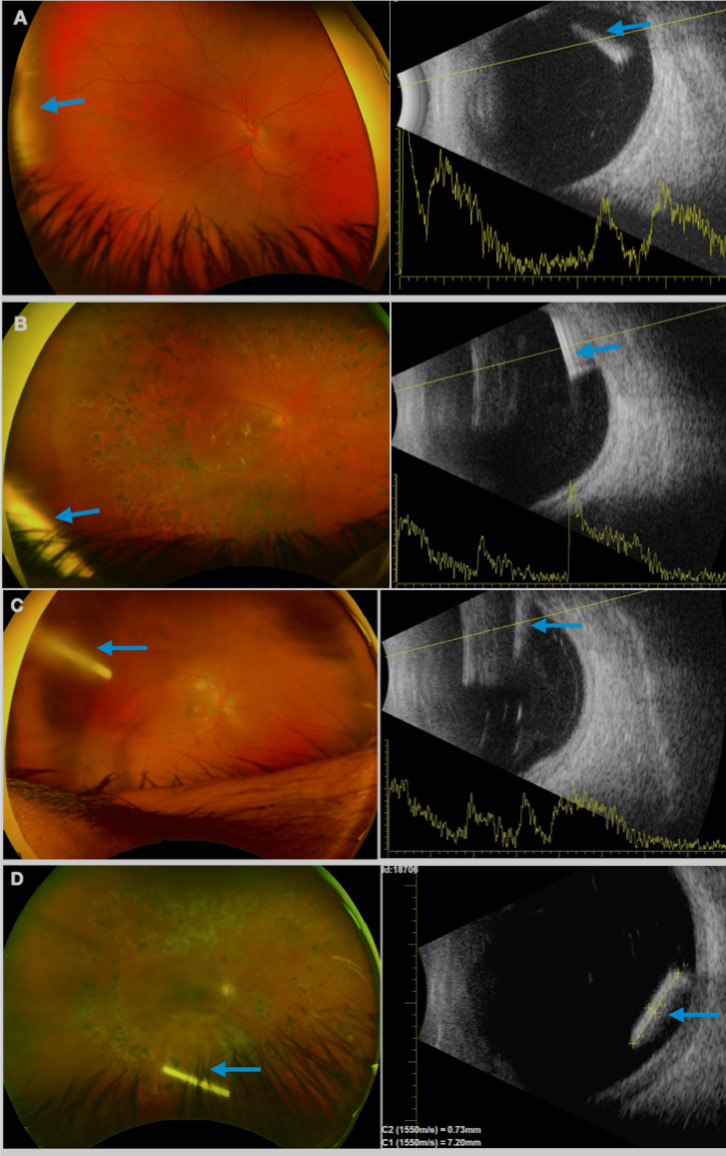
(**A**, **B**, **C** and **D**) Ultra-widefield fungus photography (left) and B-scan (right) showing Ozurdex^®^ implant (blue arrows) correlated location, in D0 visits of different eyes

*Ultra-widefield fundus photograph* Seventy-nine percent of the implants could be visualized in the ultra-widefield fundus image. Common difficulties proper to the method, such as the sizes of the eyelid, nose, and pupil and difficulty maintaining the eye open may explain the lack of implant identification by the ultra-widefield photos. In all the cases where visualization was possible, a positive correlation with B-scan finding was performed (Fig. 6A, B, C, and D), despite the change in patient position (reclined for the B-scan and seated for ultra-widefield fundus photography).

## Discussion

Intravitreal drugs are widely used and are preferred over topical and intravenous applications, due to the proximity through which the drug can reach the vitreous cavity, retina, and choroid [3, 4]. The possibility to merge nanoparticles of a target drug into a polymer delivery system permits its long and slow release [5–7]. Hydrolysis and autocatalysis start when the Ozurdex® implant reaches the vitreous cavity and works independently of the process of drug release [6]. It is known that an “empty” polymer structure may be found in the vitreous cavity when the drug is not more effective or even when other new implants need to be injected [8].

Although the Ozurdex^®^ implant’s functionality is well documented [9–11], its structural characteristics over time in human eyes, such as movement, location, relationship with intraocular tissues, and how decomposition occurs, are not documented to the best of our knowledge. Case reports enrolling Ozurdex^®^ implants located close to the posterior pole and studied with optical coherence tomography [12–14] could reveal aspects close to our findings: initially a dense and cylindrical structure, high density, and hyper-reflectivity, indicating a shadowing artifact; followed by a sequential lowering of the internal reflectivity, maintaining the external structure. Costello and cols [15] confirmed that glucose polymer degradation initiates internally, leaving a final hollow structure. It is known that ocular ultrasonography is operator-dependent and studies that associate this technique with multimodal evaluations, such as ultra-widefield fundus photography [16], help in creating objective parameters to guide and help in differentiation from other intraocular findings observed in daily practice, as foreign bodies [17, 18], lens content, clots, previous ocular medication [19], and dislocated intraocular lenses. The possibility to estimate the timing of the implantation based on B-scan aspects is useful when there is no clear media and/or no access to clinical data revealing when the procedure took place. Restrictions on elective exams during the COVID-19 pandemic affected this present study for follow-up exams; however, the findings obtained could be studied independently, once there was no evidence of inter-individual variability in the evaluated parameters.

## Conclusion

The present study revealed a progressive decrease in the implant measurements (especially in length) at follow-up, as observed by ocular ultrasonography and ultra-widefield fundus photography. Qualitative changes in the fading and lowering of internal reflectivity were detected over time. These alterations evidenced in the Ozurdex® implant over time may be related to the degradation of the glucose polymer structure. Nevertheless, we recommend that more multicenter studies with larger study samples should be conducted to further investigate our findings.

## Data Availability

No datasets were generated or analysed during the current study.

## Abbreviations

USA: United States of America
mg: Milligrams
mm: Millimeters
